# Native Quercetin as a Chloride Receptor in an Organic Solvent

**DOI:** 10.3390/molecules23123366

**Published:** 2018-12-19

**Authors:** Mohamed Lamin Abdi Bellau, Olga Bortolini, Giancarlo Fantin, Marco Fogagnolo, Daniele Ragno, Ignacio Delso, Pedro Merino

**Affiliations:** 1 Dipartimento di Scienze Chimiche e Farmaceutiche, Università di Ferrara, Via Luigi Borsari 46, I-44121 Ferrara, Italy; mohamedlamin.abdibellau@unife.it (M.L.A.B.); olga.bortolini@unife.it (O.B.); marco.fogagnolo@unife.it (M.F.); daniele.ragno@unife.it (D.R.); 2 Instituto de Sintesis Quimica y Catalisis Homogenea (ISQCH). Universidad de Zaragoza-CSIC, 50009 Zaragoza, Spain; 3 Instituto de Biocomputación y Física de Sistemas Complejos (BIFI), Universidad de Zaragoza, 50009 Zaragoza, Spain; pmerino@unizar.es

**Keywords:** quercetin, chloride binding, NMR titrations, quantum mechanics density functional theory (QM-DFT) calculations, molecular dynamics (MD) calculations

## Abstract

The binding properties of quercetin toward chloride anions were investigated by means of ^1^H-NMR, ^13^C-NMR, and electrospray ionization mass spectrometry (ESI-MS) measurements, as well as computational calculations. The results indicate that quercetin behaves primarily as a ditopic receptor with the binding site of the B ring that exhibits stronger chloride affinity compared to the A ring. However, these sites are stronger receptors than those of catechol and resorcinol because of their conjugation with the carbonyl group located on the C ring. The 1:1 and 1:2 complexation of this flavonoid with Cl^−^ was also supported by ESI mass spectrometry.

## 1. Introduction

The search for anion receptors is an important area of supramolecular chemistry due to its importance and potential applications in a wide range of chemical and biological processes [1,2,3,4,5]. Most of these receptors have N–H or N–H^+^ groups contributing to bind anions through H-bonding and/or electrostatic interactions. In other cases, metals or Lewis acids can also act as binding sites [6]. In this framework, small natural products capable of binding and transporting anions, specifically chloride, across hydrophobic membranes are relatively few [7]. The growing interest in molecules that bind and “chaperone” the chloride anion is inspired, in large part, by the knowledge that a number of diseases are caused by the misregulation of this anion across cell membranes [8]. With an eye toward the identification of naturally occurring molecules [9] that could potentially carry out the abovementioned functions, we considered quercetin as a candidate. In fact, this compound possesses hydroxyphenyl moieties for the anion recognition [10,11] and shows affinity to hydrophobic phases such as lipid membranes [12,13]. These considerations prompted us to investigate the binding properties of quercetin toward halides in non-competitive solvents. Moreover, despite many studies on this molecule as ligands for metal cations [14,15,16,17,18,19,20], to our knowledge, there are no reports concerning interactions of quercetin with anions.

Quercetin is a naturally occurring polyphenol aglycone, belonging to a group of phytochemicals called polyphenol flavonoids, present in a variety of vegetables and fruits, such as onions, apples, berries, broccoli, tea, and red wine, and is considered a health protective phytonutrient [21].

This molecule is, in fact, a potent antioxidant and oxygen radical scavenger, and it is also recognized for having antibacterial, antiviral, antitumoral, and anti-inflammatory properties [22,23,24].

**Figure 1:**
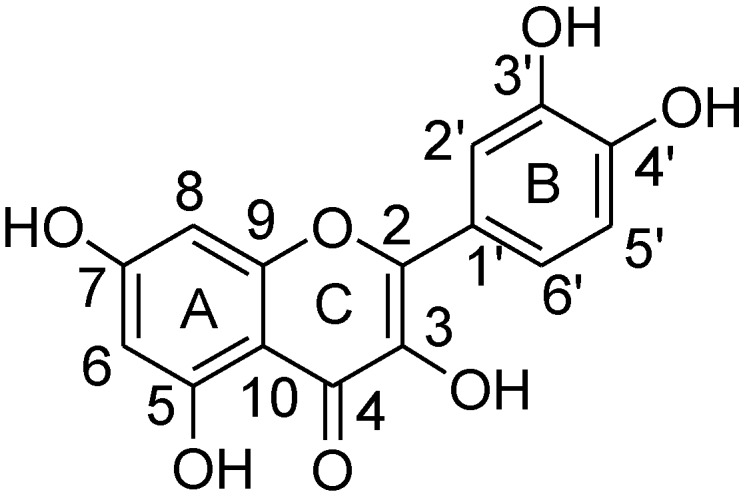
Quercetin (**1**).

Quercetin is a 3,5,7,3′,4′-pentahydroxy phenol, having a resorcinol-type arrangement of hydroxyl groups in the A ring and a catechol-type structure in the B ring (Figure 1). In the C ring, quercetin also contains a 2,3-double bond and 4-oxo function which can give conjugation with the adjacent aromatic rings. 

## 2. Results and Discussion

When solid methyltrioctylammonium chloride was added to a solution of quercetin **1** in deuterated acetone, the chemical shifts of the corresponding ^1^H-NMR spectrum showed dramatic changes. The possibility of aggregation of quercetin was negligible since the ^1^H-NMR spectra were substantially identical at concentrations within the range of 1–5 mM [25]. Consequently, the results clearly indicated an interaction between the chloride anion and this flavonoid. 

### 2.1. ^1^H-NMR Binding Studies

**Figure 2:**
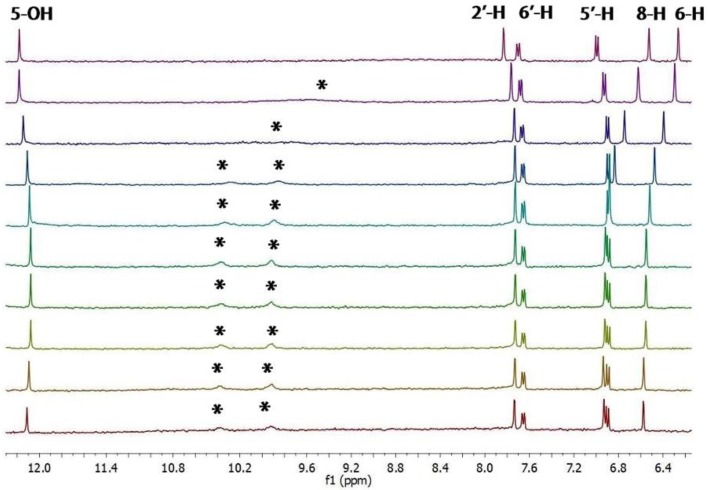
Variations of a portion of ^1^H-NMR spectrum (400 MHz) of quercetin (**1)** (concentration = 2.05 mM) during its titration with chloride. The molar fractions of the guest were 0, 1.16, 2.3, 3.4, 4.5, 6.6, 10.7, 16.2, and 27.1 from top to bottom. * indicates OH groups.

In order to gather information on the sites and the strength of binding we carried out an ^1^H-NMR titration of quercetin with chloride (Figure 2).

**Figure 3:**
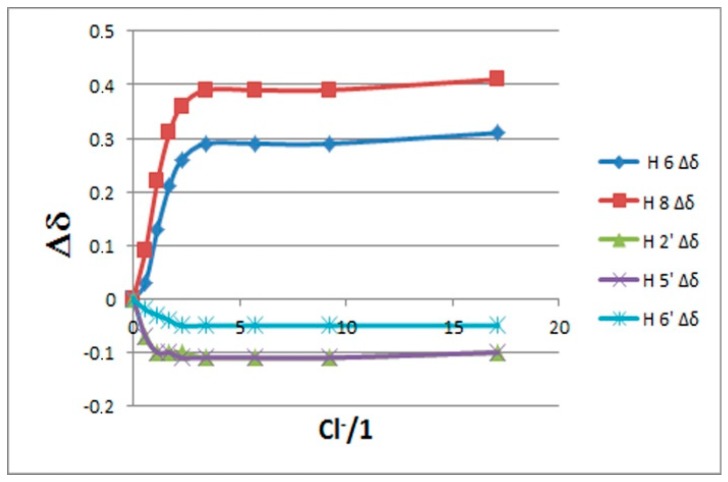
Chemical shifts change of the Ar–H protons of quercetin with increasing Cl^−^ concentration. Positive values mean downfield shifts.

The binding isotherms of the C–H protons showed an upfield shift (with hyperbolic trend) for the B-ring protons and a simultaneously more pronounced downfield shift (with sigmoidal shape) for those of the A ring (Figure 3). The behavior of the latter suggests the presence of supplementary C–H---chloride hydrogen-bonding interactions in the A ring [26]. The NMR signals of the OH protons, with the exception of 5-OH, were indistinguishable at the beginning of titration [27]. On the other hand, they became visible during titration by moving downfield (see Figure 2). This fact suggests that the interaction between the chloride anion and these phenolic OH groups occurs without deprotonation.

The simultaneous fitting (HypNMR 2008 program [28]) of the saturation curves of Figure 3 achieved by applying both 1:1 and 1:2 binding model gave binding constants with very large uncertainties (>200%), suggesting that these results are meaningless. More satisfactory results were obtained considering quercetin as a ditopic receptor and fitting the isotherms of the A and B ring separately using a 1:1 binding model [29]; the corresponding binding constants (as Log K values) are reported in Table 1.

The binding constant obtained for the B ring (catechol moiety) was found to be surprisingly higher than the analog of the catechol (4.59 vs. 3.36; see Table 1). This is probably due to the fact that the B ring of quercetin is conjugated to the C ring via an α,β-unsaturated carbonyl moiety, and this leads to an increase in the acidity of the 4’-OH group which is responsible, together with the 3’-OH group, for the coordination of the chloride anion in a chelate mode [10].

**Figure 4:**
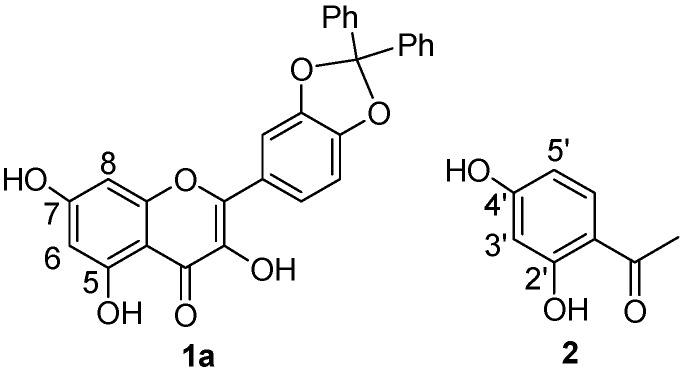
Quercetin diphenylmethylketal **1a** and 2′,4′-dihydroxyacetophenone **2**.

**Figure 5:**
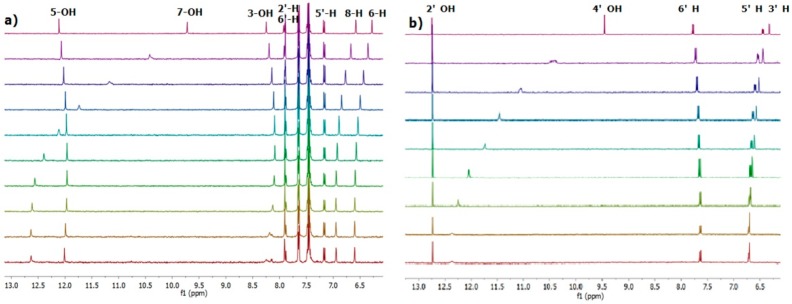
(**a**) Variations of a portion of ^1^H-NMR spectrum (400 MHz) of **1a** (2 mM) during its titration with chloride. The molar fractions of the guest were 0, 0.99, 1.96, 2.91, 3.85, 5.66, 9.09, 23.08, 28.86, and 34.31 from top to bottom. (**b**) Variations of a portion of ^1^H-NMR spectrum (400 MHz) of **2** (2 mM) during its titration with chloride. The molar fractions of the guest were 0, 1.02, 2.02, 2.99, 3.95, 5.83, 9.36, 14.19, and 23.75 from top to bottom.

**Figure 6:**
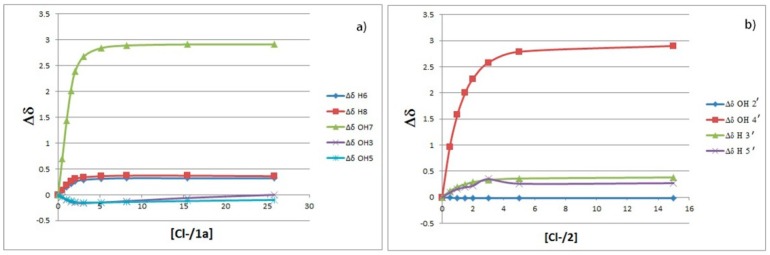
(**a**) Change in ^1^H chemical shifts of **1a** with increasing Cl^−^ concentration. (**b**) Change in ^1^H chemical shifts of **2** with increasing Cl^−^ concentration. Positive values mean downfield shifts.

Anion binding aptitude of the resorcinol moiety (A ring) of quercetin toward the chloride anion is particularly interesting; in this case, in fact, the 5-OH group is “deactivated”, due to intramolecular H-bonding with carbonyl group (C=O) [30], and only the 7-OH group remains available to bind the chloride anion. This feature is clearly verifiable in Figure 2 showing the signal of 5-OH practically unaffected throughout the whole titration. To corroborate this behavior, we performed NMR titrations of quercetin diphenylmethylketal **1a** and of 2′,4′-dihydroxyacetophenone **2**—the first compound containing only the binding site related to the resorcinol moiety and the second one having structural similarities with the A ring of quercetin (Figure 4 and Figure 5). The corresponding binding isotherms, depicted in Figure 6, have a very similar appearance; both the hydroxyl protons *ortho* to carbonyl group (5-OH of **1a** and 2’-OH of **2**) remained unaffected during titration, while the signals of the second OH (7-OH of **1a** and 4’-OH of **2**) moved decidedly downfield (this behavior is particularly evident in Figure 5). The signals of the aromatic protons vicinal of the OH groups (6-H and 8-H of **1a** and 3’-H, 5’-H of **2**) moved downfield by nearly the same magnitude for both receptors; these very significant shifts (between 0.27 and 0.38 ppm, respectively) are indicative of the co-presence of Ar–H----chloride hydrogen-bonding interactions. Moreover, the 3-OH group of the receptor **1a** was not significantly perturbed in the ^1^H-NMR titration, probably due to an intramolecular hydrogen bond with the carbonyl group, stable enough to withstand the chloride action (Figure 5a).

Data analysis fitted the 1:1 binding profile and gave a Log K of 3.02 for receptor **1a** and of 3.1 for receptor **2**.

In order to have a point of reference, binding constants of catechol and resorcinol (2 mM) in deuterated acetone and methyltrioctylammonium chloride as titrants were also carried out. The magnitude of these constants, Log K = 3.36 M^−1^ for the catechol and Log K = 2.39 M^−1^ for the resorcinol, were consistent with the results of Smith (in CD_3_CN) [10].

It is interesting to note that **1a** and **2** were found to be more effective receptors than the resorcinol probably due to a –M mesomeric effect of the carbonyl group engaged in a intramolecular C=O⋯H−O hydrogen bond that increases the acidity of the *para* OH group, as well as that of the adjacent aromatic protons. Indeed, it was reported that the pK_a1_ value for resorcinol is 9.5 [31], while that calculated for 2′,4′-dihydroxyacetophenone (**2**) is significantly lower at 7.9 [32]. A lower pK_a_ value is consistent with a greater capability of acting as hydrogen-bond donors. 

The binding attitude of quercetin to bromide was also studied (for details, see Supplementary Materials). As expected [10], the binding strength of quercetin for bromide (β_1_ = 1.62 M^−1^; β_2_ = 1.61 M^−1^) was significantly smaller than that displayed toward chloride (see Table 1). It is interesting to note that catechol moiety showed a significant degree of dimensional selectivity for the chloride and bromide anions (β_1_ = 4.59 vs. 1.62 M^−1^, respectively), about three orders of magnitude. In the case of the resorcinol moiety, however, the chloride was only bound a factor of ca. 10 stronger than bromide (β_1_ = 2.95 vs. 1.61 M^−1^, respectively), thus indicating a much lower degree of size selectivity. Similar trend of dimensional selectivity for the chloride and bromide anions, although less pronounced, was also observed for the individual catechol and resorcinol molecules [10].

### 2.2. ^13^C-NMR and ESI-MS Binding Studies

**Figure 7:**
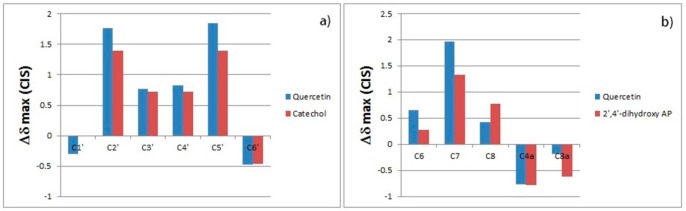
(**a**) Δδ_max_ of the carbons of the B ring of quercetin compared with analogous carbons of catechol after addition of an excess of chloride anion. (**b**) Δδ_max_ of carbons of the A ring of quercetin compared with analogous carbons of 2′,4′-dihydroxyacetophenone after addition of an excess of chloride anions. Positive values mean downfield shifts. All spectra were run at a concentration of 20 mM.

We compared the maximum perturbation caused by an excess (25 equiv.) of chloride anion on the ^13^C-NMR chemical shifts of quercetin, catechol, and 2′,4′-dihydroxyacetophenone (20 mM in acetone) (Figure 7). Figure 7a shows that the complexation-induced shifts (CISs) on the B-ring carbons of quercetin and on the carbons of the structurally analogous catechol have a very similar trend; therefore, it can be stated that very similar bond interactions occur between the chloride and each receptor. Similar CISs were also observed for the carbons belonging to the A ring of quercetin and for the analogous carbons of 2′,4′-dihydroxyacetophenone (Figure 7b). This indicates that the interactions of the chloride anion with the A ring of the quercetin and the 2′,4′-dihydroxyacetophenone are very similar.

Binding interactions between quercetin and chloride anion were also confirmed by electrospray negative-ion mass spectrometry (ESI-MS) which revealed a peak corresponding to the 1:1 complex (**1**⋯Cl^−^) at *m*/*z* 337 (25%), and a base peak at *m*/*z* 740 relative to the 1:2 complex (Me(Octyl)_3_N^+^Cl^−^⋯**1**⋯Cl^−^) (See Figure S9, Supplementary Materials).

### 2.3. Molecular Modeling

**Figure 8:**
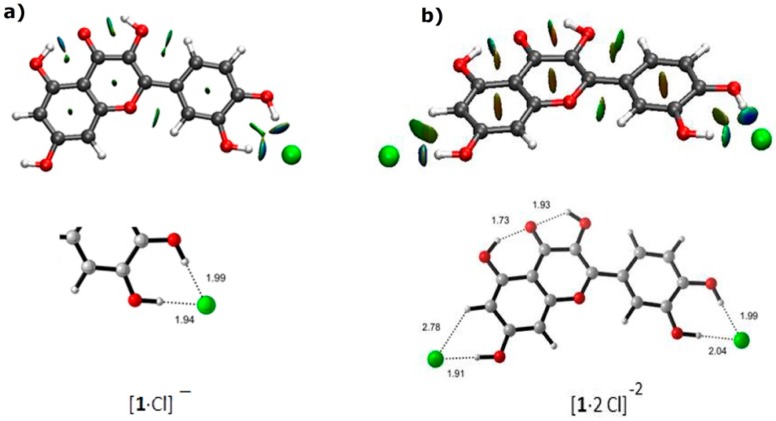
Optimized geometries, showing non-covalent interaction (NCI) surfaces: (**a**) 1:1 complex of chloride anion with quercetin; (**b**) 2:1 complex of chloride anion with quercetin. Details are given with distances indicated in ångström.

The complexes of quercetin with chloride anion were examined using density functional theory (DFT) calculations (for details, see Supplementary Materials). The structure of quercetin was minimized. Then we studied 1:1 chloride complexes, and three relative energy minima were found for each structure in which the chloride anion was positioned at the resorcinol moiety, at the catechol moiety, and at the proximities of the 3-hydroxyl group. The most stable geometry corresponded to the complex where the chloride anion was located at the catechol moiety, predicting 100% of abundance due to the observed difference in energy. The optimized geometry of this minimum is shown in Figure 8a. In the case of 2:1 chloride complexes, a unique minimum with chloride anions located at the resorcinol and catechol moieties was found (Figure 8b). Strong directional interactions were observed between chloride anions and hydroxyl groups in agreement with spectroscopic observations. Non-covalent interaction (NCI) analyses confirmed such interactions as typical green-blue discs featuring strong non-covalent interactions. 

**Figure 9:**
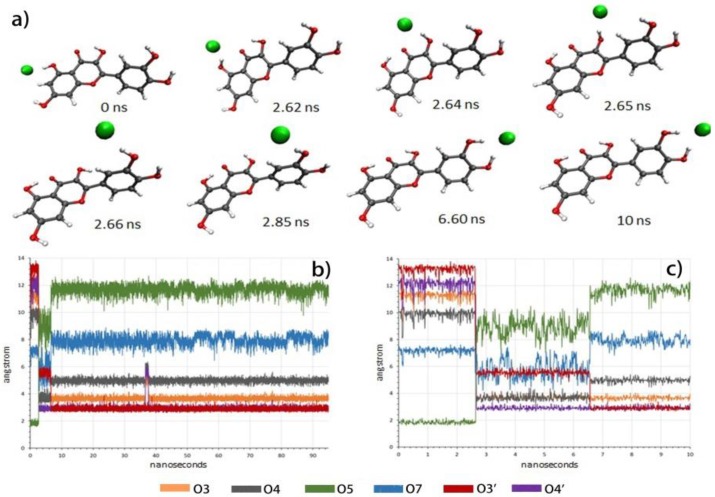
(**a**,**b**) Interaction distances between oxygen atoms and chloride anions during the simulation starting from the chloride anion located at the resorcinol moiety; (**c**) expansion of the first 10 ns of subfigure (**b**).

To study the dynamics of the recognition process, molecular dynamics (MD) simulations were performed with AMBER16 and AMBERTools16. Relevant interaction distances between oxygen atoms and chloride anions were followed during the simulation (Figure 9).

When the MD started, considering the complex where the chloride anion was placed at the resorcinol moiety, the system evolved and a snapshot taken after 2.65 ns showed the chloride in the proximity of O3. Further snapshots showed the movement of chloride toward the catechol and, after 6.60 ns, the complex at the catechol was raised. As expected, when the chloride anion was placed at the catechol moiety, it remained in the proximity of O3’ and O4’, reflecting the stability of the complex. These results clearly established the recognition by the catechol moiety as the most stable, in agreement with experimental observations. 

With a second chloride anion, the situation remained completely stable with chloride anions located at resorcinol and catechol moieties (See Figure S12, Supplementary Materials), as predicted by DFT calculations and in good agreement with experimental observations.

## 3. Materials and Methods

### 3.1. General Comments

All the reagents and solvents used in this study were bought from commercial sources. The NMR spectra were recorded in acetone-*d*_6_ solvent using 5-mm tubes, at 298 K, with a Varian Mercury Plus 400, (Varian Inc., Palo Alto, CA, USA) operating at 400 (^1^H) and 100 MHz (^13^C). The chemical shifts were referenced to acetone: δ (H) 2.04 ppm and δ (C) 29.0 ppm. Some ^13^C-NMR spectra were acquired in non-deuterated acetone; in this case, a capillary filled with C_6_D_6_ (secondary standard [33]) was placed into the 5-mm tube and the sample was locked and shimmed on C_6_D_6_ contained in the secondary standard, and the chemical shifts were also referenced to C_6_D_6_: δ (C) 127.6 ppm. ESI mass spectra were obtained using an LCQ Duo (ThermoQuest, San Jose, CA, USA) in negative-ion mode. Instrumental parameters were as follows: capillary voltage −10 V, spray voltage 4.50 kV, mass scan range was from *m*/*z* 100 to 2000 amu, for 30,000 ms of scan time; N_2_ was used as sheath gas. The samples were injected into the spectrometer through a syringe pump at a constant flow rate of 8 mL/min. 

### 3.2. ^1^H-NMR Titration

The following is a typical procedure for ^1^H-NMR titration: 1 mL of a 2 mM solution of host in undried acetone-*d*_6_ [34] was placed in a 5-mm NMR tube and an initial spectrum was taken. A measured amount of a 100 mM solution of guest (as methyl trioctylammonium salt) in the same solvent was added, changing the molar fraction of guest to about 0, 0.5, 1, 1.5, 2, 3, 5, 10, 20, and 30. Spectra were recorded after each addition. The chemical shift variation of the host signals was collected and the binding constants β (as Log K) were calculated with the curve-fitting method [35] using the commercial HypNMR2008 [28] program (details are given in Supplementary Materials).

## 4. Conclusions

In summary, the anion-binding properties of the natural flavonoid quercetin in an organic solvent for chloride anions were studied using spectroscopic (NMR and MS) and computational (DFT and MD) methods.

We demonstrated that quercetin, in the NMR titrations, behaves as a ditopic receptor at the A and B rings; in fact, no significant shifts of the C ring protons occur. It is interesting to note that the affinity of the resorcinol moiety of quercetin and of its diphenymethylketal derivative **1a** for chloride anions is found to be similar to that of 2′,4′-dihydroxyacetophenone **2**, which in turn is greater than that of simple resorcinol. Furthermore, the bond constant obtained for the B ring (catechol moiety) is surprisingly high, about seventeen times greater than the catechol analog. This is probably due to the fact that the B ring of quercetin is conjugated to the C ring via an α,β-unsaturated carbonyl group. 

## Figures and Tables

**Table 1:** Binding constants β (as Log K) for chloride (as methyltrioctylammonium salt) by hosts **1**–**2**, resorcinol and catechol in acetone-*d*_6_ at 298 K ^a^.

Host	β (M^−1^) ^a^ from CH Protons	β (M^−1^) ^a^ from OH Protons
Quercetin (**1**)	β_1_ = 4.59 ^b^ (error = 36%) β_2_ = 2.95 ^b^	n.d. ^c^
**1a**	β_1_ = 3.01 ^b^ β_2_ n.d.	β_1_ = 3.02 ^b^ β_2_ n.d.
**2**	β = 3.09	β = 3.10
Catechol	β = 3.36	β = 3.29
Resorcinol	β = 2.39	β = 2.12

^a^ Estimated errors ≤10% unless otherwise stated. ^b^ Binding events on the host were fitted separately as a 1:1 single episode. ^c^ Not determined due to signals overlapping.

## Supplementary Materials

The supplementary materials are available online.
