# Supplementary material for: Native Quercetin as a Chloride Receptor in an Organic Solvent
Source: Molecules. 2018 Dec 19;23(12):3366. doi: 10.3390/molecules23123366 (PMC6320934; doi:10.3390/molecules23123366)
Supplement: Supplementary file 1 [file molecules-23-03366-s001.pdf]

Native quercetin as chloride receptor in organic solvent

Mohamed Lamin Abdi Bellau [1], Olga Bortolini [1], Giancarlo Fantin [1,*], Marco Fogagnolo [1], Daniele Ragno [1], Ignacio Delso [2,*] and Pedro Merino [3]

[1] Dipartimento di Scienze Chimiche e Farmaceutiche, Università di Ferrara, Via Luigi Borsari 46, I-44121 Ferrara, Italy; mohamedlamin.abdibellau@unife.it (M.L.A.B.); olga.bortolini@unife.it (O.B.); marco.fogagnolo@unife.it (M.F.); daniele.ragno@unife.it (D.R.)

[2] Instituto de Sintesis Quimica y Catalisis Homogenea (ISQCH). Universidad de Zaragoza-CSIC, 50009 Zaragoza, Spain

[3] Instituto de Biocomputación y Física de Sistemas Complejos (BIFI), Universidad de Zaragoza, 50009 Zaragoza, Spain; pmerino@unizar.es

* Correspondence: fnn@unife.it (G.F.); idelso@unizar.es (I.D.); Tel.: +039-0532-455946 (G.F.)

Supporting Information

Titration data and calculation of binding constants **2**

ESI-MS measures **18**

Computational Methods **19**

Geometries **20**

Molecular dynamics **21**

References **22**

^{1}H NMR titration

The following is a typical procedure for 1H NMR titration: 1 mL of a 2 mM solution of host in acetone-d6 was placed in a 5 mm NMR tube and an initial spectrum was taken. A measured amount of a 100 mM solution of guest (as methyl trioctylammonium salt) in the same solvent was added changing the molar fraction of guest to about 0, 0.5, 1, 1.5, 2, 3, 5, 10, 20, 30. Spectra were recorded after each addition. The chemical shift variation of the host signals was collected and the binding constants (as log K) were calculated by curve fitting method [1] using the commercial HypNMR2008[2] program. Estimated errors ≤ 10% unless otherwise stated.

Titration of quercetin (1) 2 mM with chloride 100 mM in acetone-d6

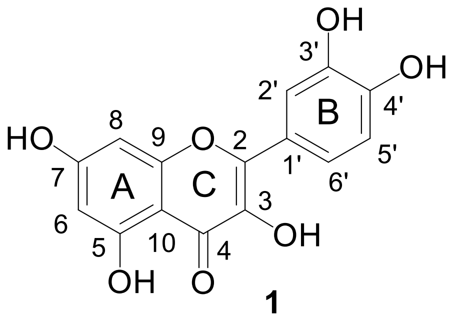

Figure S1. Quercetin

HYPNMR fitting data output for Cl^{-} binding by quercetin (**1**) calculate from H6-H8 protons (ring A).

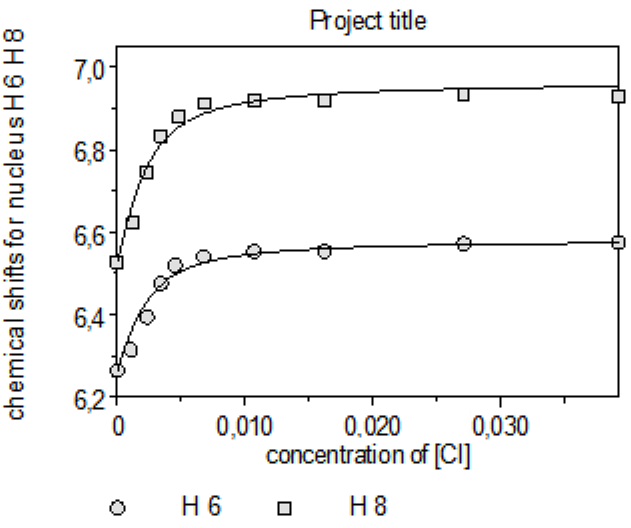

HypNMR2008

Refinement concluded at 16:34:26 on 28/02/2018

Data from C:\Users\Fantin\Desktop\Q-Cl 1H (H6-H8).

Project title: TITRATION OF QUERCETIN + CHLORIDE RING A by 1H NMR

Converged in 5 iterations with sigma = 2,852036

	value	standard deviation	Comments
1 log beta([Cl][Q])	2.9492	0.0964	2.95(1)

HYPNMR fitting data output for Cl^- binding by quercetin (**1**) calculate from H2′-H5′-H6′ protons (ring B).

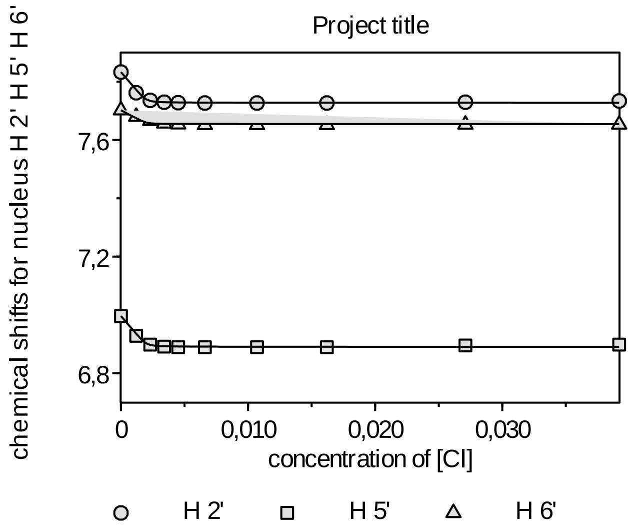

HypNMR2008

Refinement concluded at 16:52:17 on 28/02/2018

Data from C:\Users\Fantin\Desktop\Q-Cl 1H (H2'-H5'-6').

Project title: TITRATION OF QUERCETIN + CHLORIDE RING B by 1H NMR

Converged in 8 iterations with sigma = 4,143066

	value	standard deviation	Comments
1 log beta([Cl][Q])	4.5958		relative error on beta = 36%

Titration of quercetin (1) 2 mM with bromide 100 mM in acetone-d6

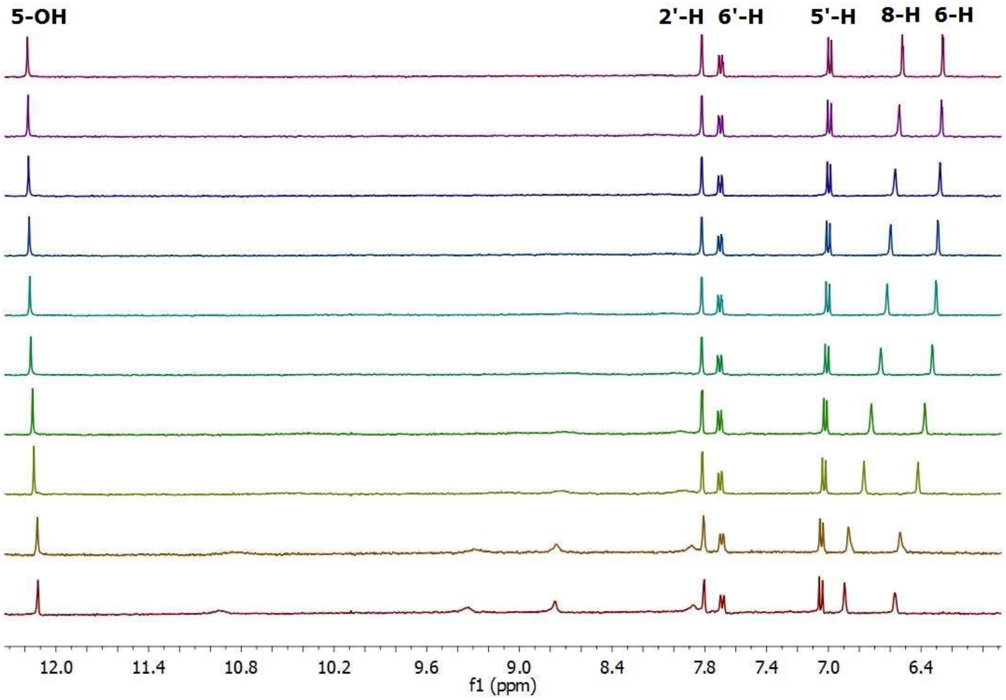

Figure S2. Variations of a portion of ^{1}H NMR spectrum (400 MHz) of Quercetin (**1**) (concentration = 2.99 mM) during its titration with Bromide. The molar fractions of guest are 0, 0.34, 0.68, 1.03, 1.34, 2.05, 3.42, 5.47, 10.27, 17.11 from top to bottom.

HYPNMR fitting data output for Br^- binding by quercetin (**1**) calculate from H6-H8 protons (ring A).

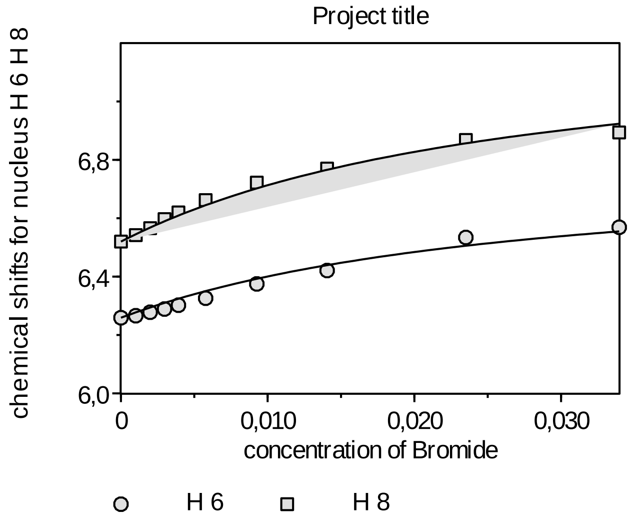

HypNMR2008

Refinement concluded at 17:48:30 on 12/11/2018

Data from C:\Users\Fantin\Desktop\QUERCETIN\HYPERNMR BUONO\Q + Br by 1H NMR\(B) Quer Bromuro 1H Ring A.

Converged in 4 iterations with sigma = 17,928866

	value	standard deviation	Comments
1 log beta(BromideQ)	1.609	0.084	1.61(8)

HYPNMR fitting data output for Br^{-} binding by quercetin (**1**) calculate from H2′-H5′-H6′ protons (ring B).

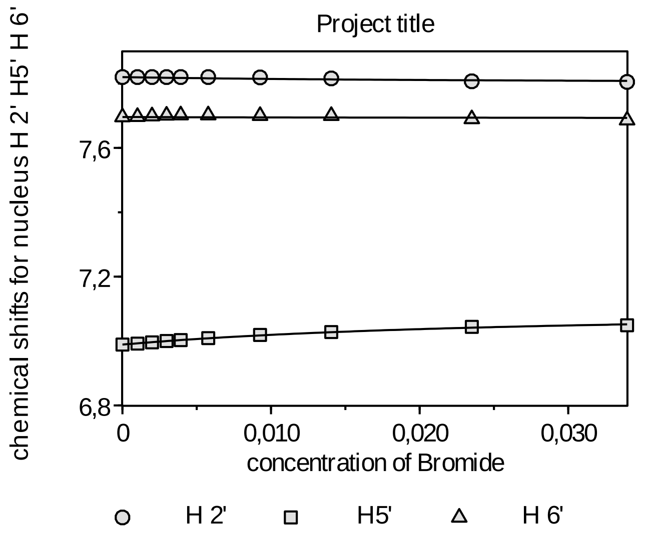

HypNMR2008

Refinement concluded at 17:42:45 on 12/11/2018

Data from C:\Users\Fantin\Desktop\QUERCETIN\HYPERNMR BUONO\Q + Br by 1H NMR\(B) Quer Bromuro 1H Ring B.

Converged in 4 iterations with sigma = 3,688622

	value	standard deviation	Comments
1 log beta(BromideQ)	1.6194	0.1338	1.6(1)

Titration of quercetin diphenylmethylketal (1a) 2 mM with chloride 100 mM in acetone-d6.

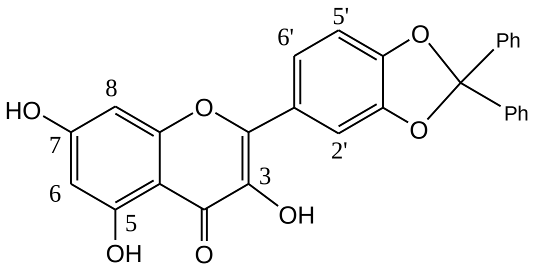

Figure S3. Quercetin diphenylmethylketal, **1a**.

HYPNMR fitting data output for Cl^- binding by quercetin diphenylmethylketal (**1a**) calculated from H6 - H8 protons (ring A).

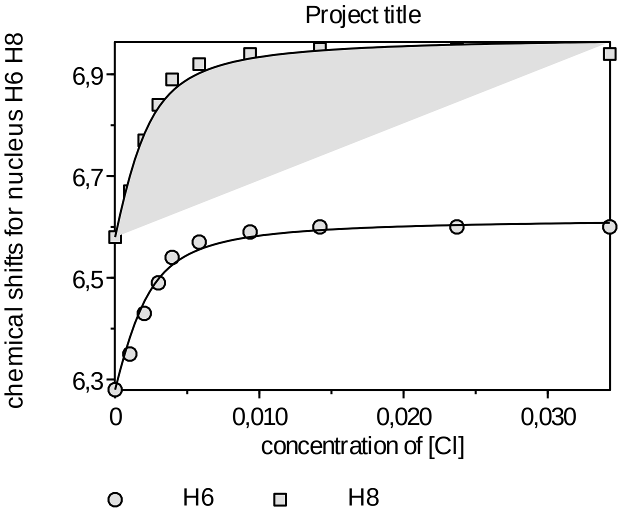

HypNMR2008

Refinement concluded at 12:12:47 on 10/07/2018

Project title: QKetal (1a) + Cl by 1H NMR \ (B) Titration QKetal-Chloride by 1H- H6,H8.

Converged in 6 iterations with sigma = 1,702190

	value	standard deviation	Comments
1 log beta([Cl][QKet])	3.0139	0.0622	3.01(6)

HYPNMR fitting data output for Cl^- binding by quercetin diphenylmethylketal (**1a**) calculated from OH7 proton (ring A).

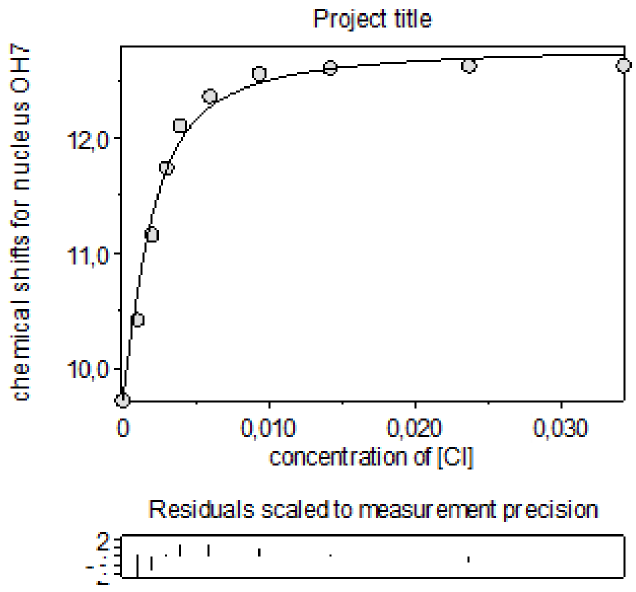

HypNMR2008

Refinement concluded at 12:49:17 on 10/07/2018

Project title: QKetal (1a) + Cl by 1H NMR \ (B) Titration QKetal-Chloride by 1H- OH7.

Converged in 5 iterations with sigma = 1,338165

	value	standard deviation	Comments
1 log beta([Cl][QKet])	3.0162	0.082	3.02(8)

Titration of 2',4'-dihydroxyacetophenone (2) 2 mM with chloride 100 mM in acetone-d6.

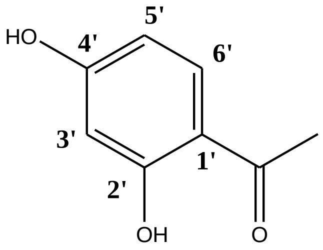

Figure S4. 2′,4′-dihydroxyacetophenone, **2.**

HYPNMR fitting data output for Cl⁻ binding by 2',4'-dihydroxyacetophenone (**2**) calculated from H3′-H5′-H6′ protons.

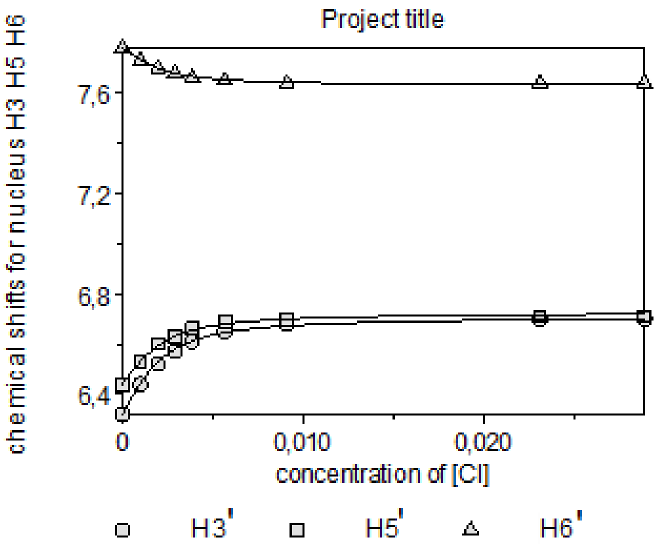

HypNMR2008

Refinement concluded at 14:26:16 on 10/07/2018

Project title: AF (2) + Cl by 1H NMR\AF + Cl 1H H- Protons.

Converged in 5 iterations with sigma = 0,567252

	standard deviation	
value		Comments

1 log beta([Cl][AF]) 3.0932 0.0232 3.09(2)

HYPNMR fitting data output for Cl⁻ binding by 2',4'-dihydroxyacetophenone (**2**) calculated from OH4′ proton.

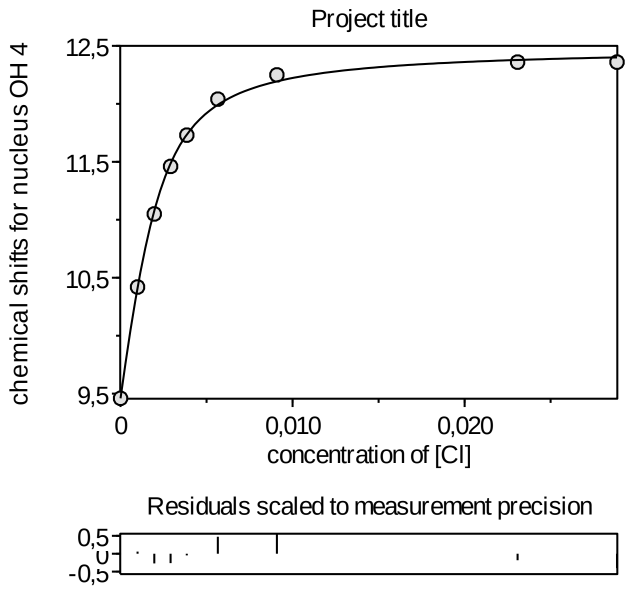

HypNMR2008

Refinement concluded at 14:30:33 on 10/07/2018

Project title: AF (2) + Cl by 1H NMR\AF + Cl 1H OH-Proton.

Converged in 5 iterations with sigma = 0,356456

	value	standard deviation	Comments
1 log beta([Cl][AP])	3.1001	0.0246	3.1(2)

Titration of resorcinol 2 mM with chloride 100 mM in acetone-d6.

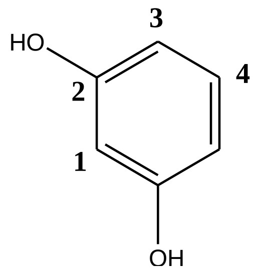

Figure S5. Resorcinol.

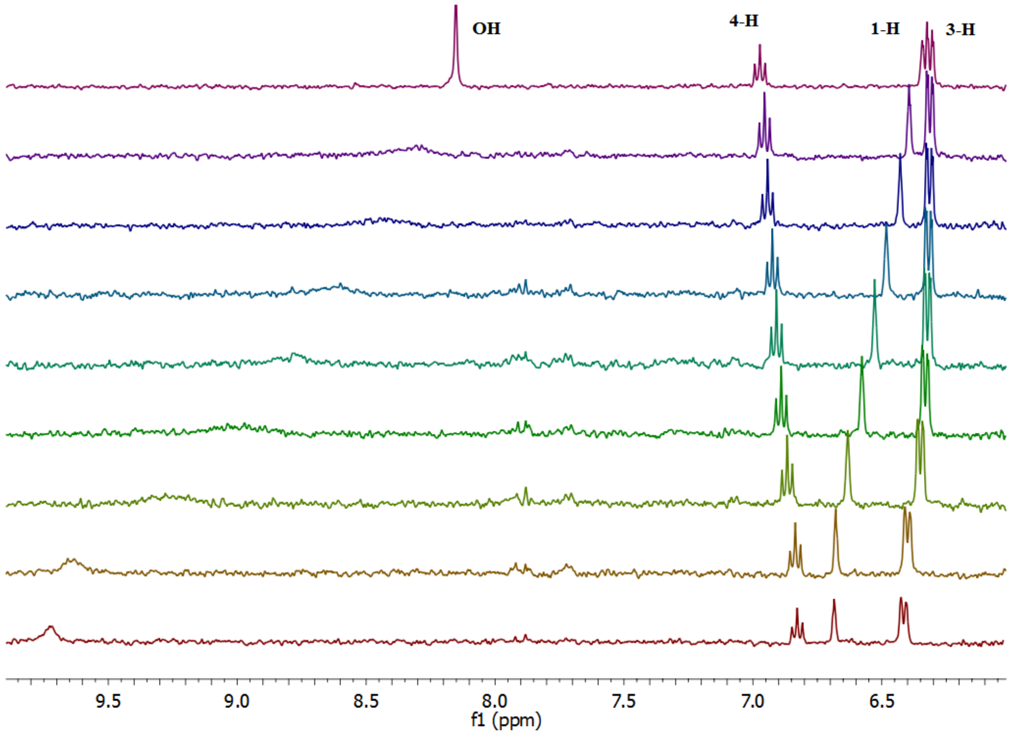

Figure S6. Variations of a portion of ^{1}H NMR spectrum (400 MHz) of Resorcinol (concentration = 2 mM) during its titration with Chloride. The molar fractions of guest are 0, 0.99, 1.96, 2.91, 3.85, 5.66, 9.09, 23.08, 28.86 from top to bottom.

HYPNMR fitting data output for Cl^- binding by resorcinol calculated from H1-H3-H4 protons.

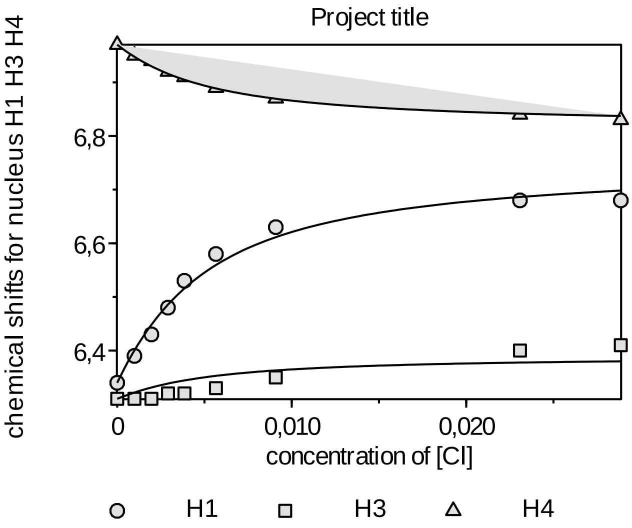

HypNMR2008

Refinement concluded at 14:38:27 on 10/07/2018

Project title: Resorcinolo + Cl by 1H NMR\Res + Cl 1H H-Protons.

Converged in 4 iterations with sigma = 1,563027

	value	standard deviation	Comments
1 log beta([Cl][Res])	2.3929	0.062	2.39(6)

HYPNMR fitting data output for Cl^- binding by resorcinol calculated from OH proton.

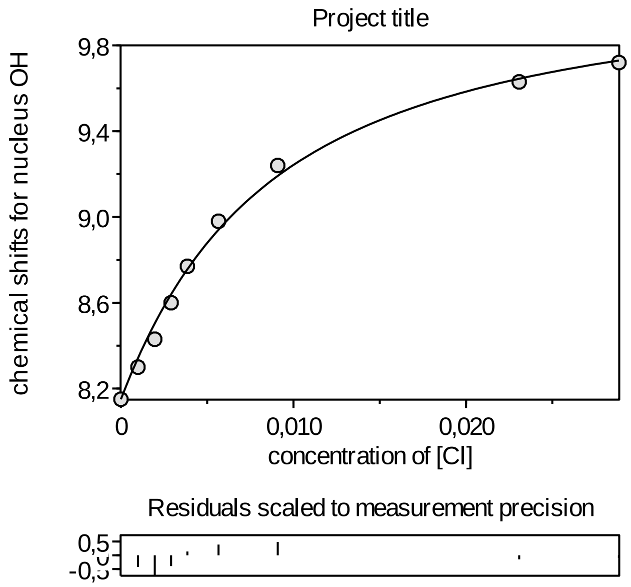

HypNMR2008

Refinement concluded at 14:46:51 on 10/07/2018

Project title: Resorcinolo + Cl by 1H NMR\Res + Cl 1H OH-Proton.

Converged in 4 iterations with sigma = 0,431397

	value	standard deviation	Comments
1 log beta([Cl][Res])	2.1198	0.0445	2.12(4)

Titration of catechol 2 mM with chloride 100 mM in acetone-d6.

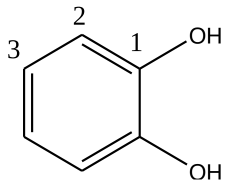

Figure S7. Catechol.

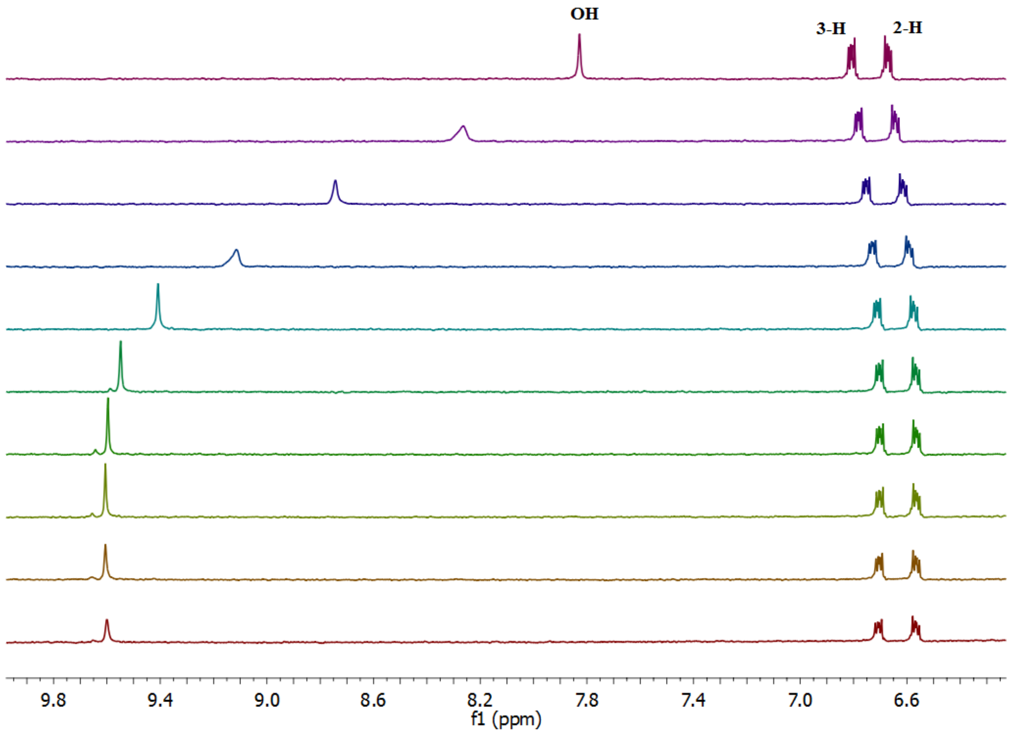

Figure S8. Variations of a portion of ^{1}H NMR spectrum (400 MHz) of Catechol (concentration = 2 mM) during its titration with Chloride. The molar fractions of guest are 0, 0.98, 1.94, 2.87, 3.81, 5.61, 9.01, 13.67, 22.87, 33.03 from top to bottom.

HYPNMR fitting data output for Cl^- binding by catechol calculated from H2-H3 protons.

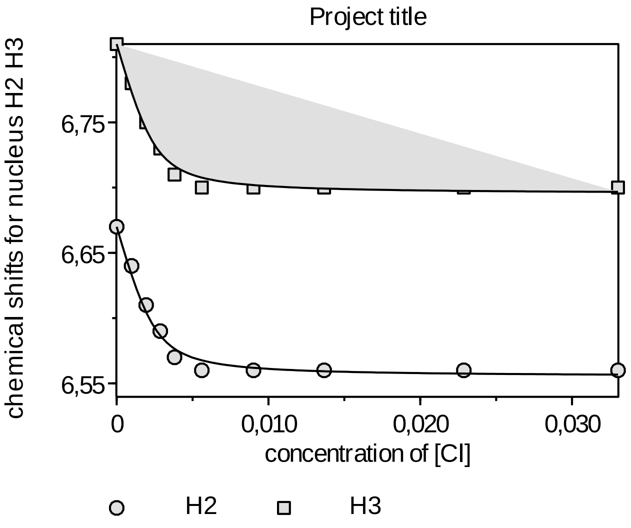

HypNMR2008

Refinement concluded at 14:58:56 on 10/07/2018

Project title: Catechol + Cl by 1H NMR\new879.

Converged in 4 iterations with sigma = 4,946284

	value	Standard deviation	Comments
1 log beta([Cl][Cat])	3.3635	0.079	3.36(8)

HYPNMR fitting data output for Cl^{-} binding by catechol calculated from OH proton.

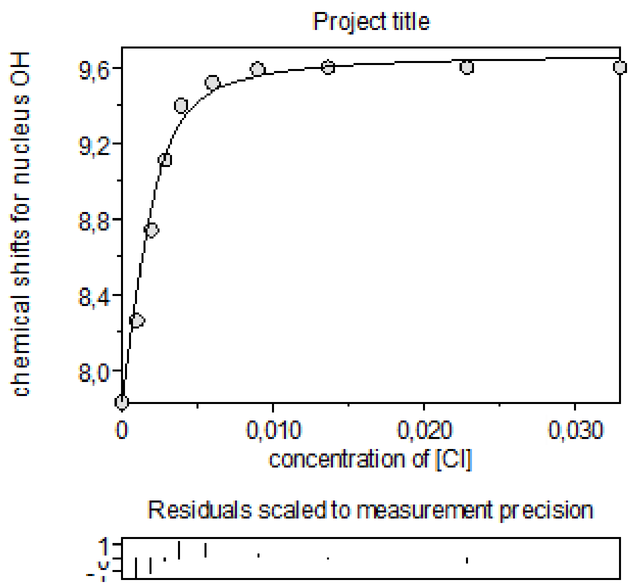

HypNMR2008

Refinement concluded at 15:05:29 on 10/07/2018

Project title: Catecolo + Cl by 1H NMR\Catecolo + Cl 1H OH.

Converged in 4 iterations with sigma = 0,870331

	value	standard deviation	Comments
1 log beta([Cl][Cat])	3.2947	0.1152	3.3(1)

ESI-MS measures

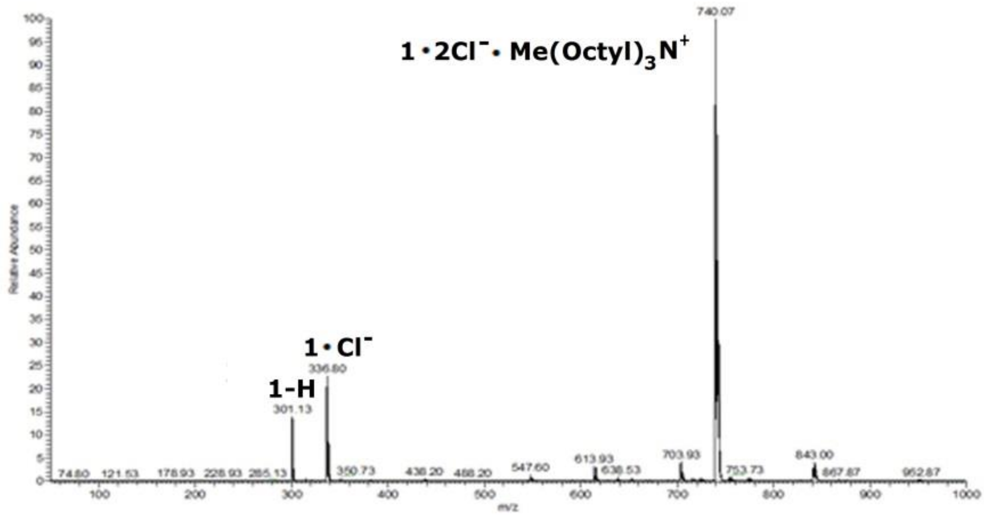

Figure S9. Negative-ion mass spectra of the CH_3CN solution containing 1 mM of salt $Me(Octyl)_3M^+Cl^-$ and 1 mM of quercetin.

Computational Methods

Geometry optimization was carried out with Gaussian09[3] using DFT methods. In particular the B3LYP functional,[4] including the D3 dispersion correction of Grimme[5] was used. The electronic configuration of the molecular systems was described with the standard split-valence basis set def2tzvp, which showed to be adequate for that functional.[6] The solvent effects were modeled as a continuum model based on the polarizable continuum model (PCM) of Tomasi's group considering acetone as a solvent.[7] Thus, the level of theory used was B3LYP-D3BJ/def2TZVP/PCM=acetone. NCI (non-covalent interactions) were computed using the methodology previously described.[8]

Data were obtained with the NCIPLOT program.[9] A density cutoff of ϱ=1.2 a.u. was applied and the pictures were created for an isosurface value of s=0.4 and colored in the [-0.3,0.3] a.u. sign(λ2)ϱ range using VMD software.[10]

Molecular dynamic (MD) simulations were carried out using AMBER 16 and AmberTools 2016.[10] The Generalized Amber Force Field (GAFF)[12] was used for the complexes parametrization and chloroform was employed as solvent, For the simulation, the complexes (best geometry from QM calculations) were placed in a rectangular box and surrounded by 1000 molecules of chloroform. Before the MD production, the system was first minimized and then relaxed over a short period to equilibrate volume and density.

The MD simulations were performed in the isothermal-isobaric ensemble (NPT) at 300 K and 1 bar, under the Periodic Boundary Conditions, using the Langevin thermostat, Berendsen barostat, non-bonded interaction cutoff set to 10 Å, no SHAKE constraints, and with a time step of 0.5 fs. The trajectory was recorded at 1-ps intervals thus resulting, as an example, in 50,000 frames for the 50-ns simulation. Analysis of the trajectory was carried out using standard tools of AMBER and VMD.[11]

Geometries

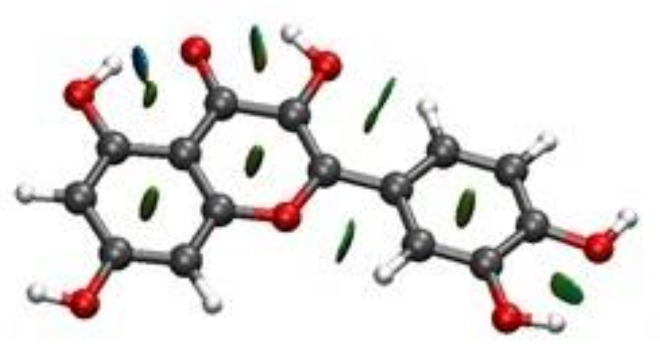

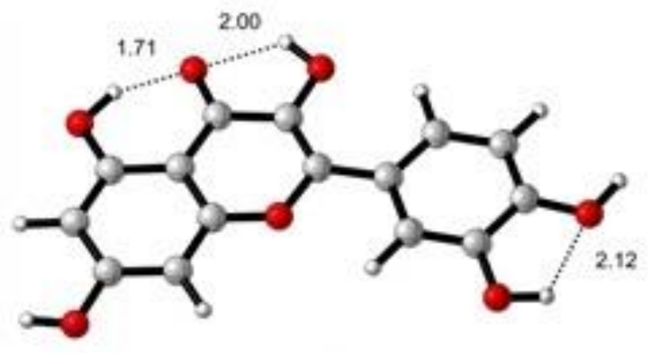

Figure S10. Top: Optimized geometries showing NCI surfaces of quercetin **1**. Bottom: Optimized geometries of quercetin **1** indicating intramolecular distances for non-covalent interactions (H-bonds).

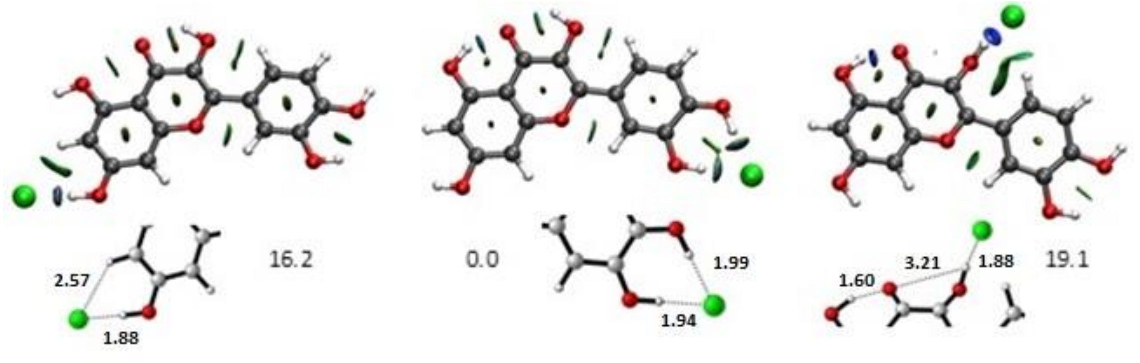

Figure S11. Optimized geometries, showing NCI surfaces, of 1:1 complexes of chloride anion with quercetin**.** Relative energies are given in kcal/mol. Details are given with distances indicated in angstrom**.**

Molecular Dynamics

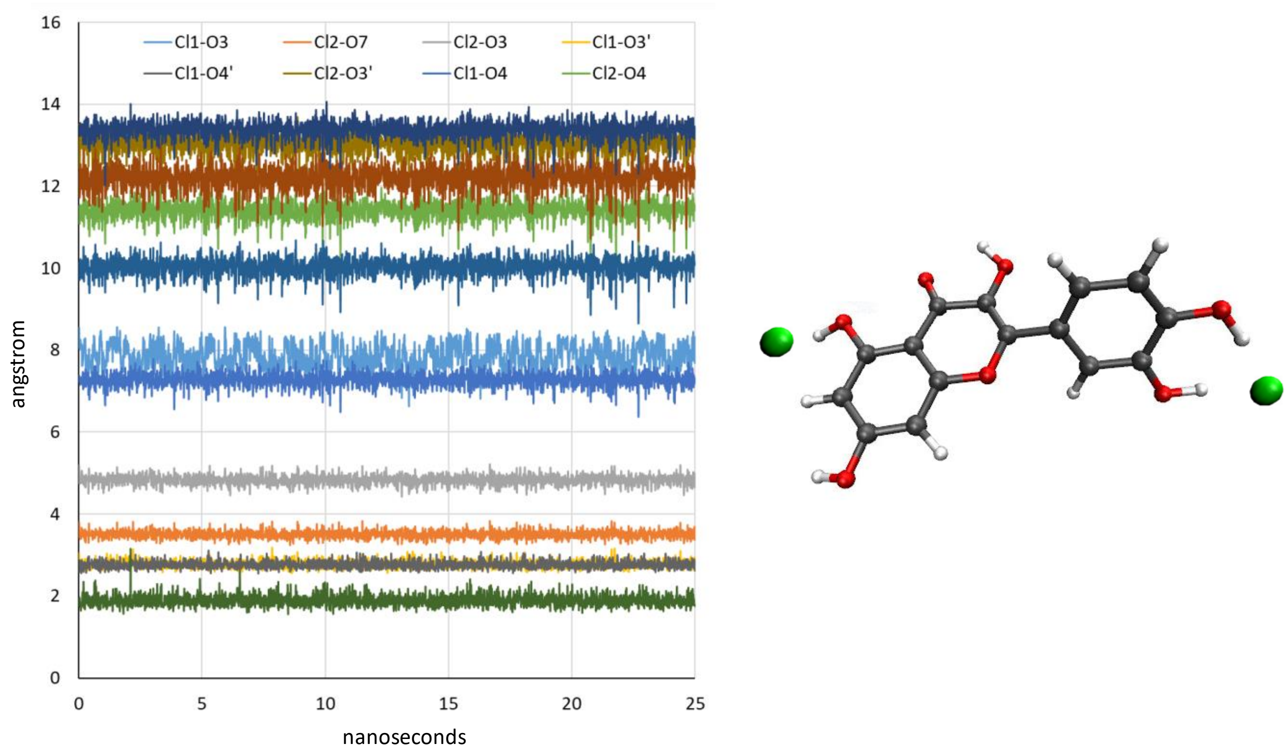

Figure S12. Molecular dynamics simulations for quercetin **1** in complex with two chloride anions located at catechol and resorcinol moieties.

References

1. Fielding, L. *Tetrahedron* 2000, 34, 6151-6170.

2. Frassineti, C.; Ghelli, S.; Gans, P.; Sabatini, A.; Moruzzi, M. S.; Vacca, A. *Anal. Biochem.* 1995, 231, 374-382.

3. Frisch, M. J.; Trucks, G. W.; Schlegel, H. B.; Scuseria, G. E.; Robb, M. A.; Cheeseman, J. R.; Scalmani, G.; Barone, V.; Mennucci, B.; Petersson, G. A.; Nakatsuji, H.; Caricato, M.; Li, X.; Hratchian, H. P.; Izmaylov, A. F.; Bloino, J.; Zheng, G.; Sonnenberg, J. L.; Hada, M.; Ehara, M.; Toyota, K.; Fukuda, R.; Hasegawa, J.; Ishida, M.; Nakajima, T.; Honda, Y.; Kitao, O.; Nakai, H.; Vreven, T.; Montgomery, J., J. A.; Peralta, J. E.; Ogliaro, F.; Bearpark, M.; Heyd, J. J.; Brothers, E.; Kudin, K. N.; Staroverov, V. N.; Kobayashi, R.; Normand, J.; Raghavachari, K.; Rendell, A.; Burant, J. C.; Iyengar, S. S.; Tomasi, J.; Cossi, M.; Rega, N.; Millam, J. M.; Klene, M.; Knox, J. E.; Cross, J. B.; Bakken, V.; Adamo, C.; Jaramillo, J.; Gomperts, R.; Stratmann, R. E.; Yazyev, O.; Austin, A. J.; Cammi, R.; Pomelli, C.; Ochterski, J. W.; Martin, R. L.; Morokuma, K.; Zakrzewski, V. G.; Voth, G. A.; Salvador, P.; Dannenberg, J. J.; Dapprich, S.; Daniels, A. D.; Farkas, Ö.; Foresman, J. B.; Ortiz, J. V.; Cioslowski, J.; Fox, D. J.; Gaussian, Inc., Wallingford CT,: 2009.

4. (a) Becke, A. D. J. Chem. Phys. 1993, 98, 5648-5652. (b) Lee, C.; Yang, W.; Parr, R. G. Phys. Rev. B 1988, 37, 785-789.

5. (a) Grimme, S. J. Comput. Chem. 2006, 27, 1787-1799. (b) Grimme, S.; Antony, J.; Ehrlich, S.; Krieg, H. J. Chem. Phys. 2010, 132, 154104-154119.

6. (a) Weigend, F. Phys. Chem. Chem. Phys. 2006, 8, 227-236. (b) Weigend, F.; Ahlrichs, R. Phys. Chem. Chem. Phys. 2005, 7, 3297-3305.

7. (a) Tomasi, J.; Persico, M. Chem. Rev. 1994, 94, 2027-2094. (b) Barone, V.; Cossi, M.; Tomasi, J. J. Comput. Chem. 1998, 19, 404-417.

8. (a) Johnson, E. R.; Keinan, S.; Mori-Sanchez, P.; Contreras-Garcia, J.; Cohen, A. J.; Yang, W. J. Am. Chem. Soc. 2010, 132, 6498-6506. (b) Lane, J. R.; Contreras-Garcia, J.; Piquemal, J.-P.; Miller, B. J.; Kjaergaard, H. G. J. Chem. Theory

Comput. 2013, 9, 3263-3266. (c) Contreras-García, J.; Calatayud, M.; Piquemal, J.-P.; Recio, J. M., Comput. Theor. Chem. 2012, 998, 193-201

9. Contreras-Garcia, J.; Johnson, E. R.; Keinan, S.; Chaudret, R.; Piquemal, J.-P.; Beratan, D. N.; Yang, W. J. Chem. Theory Comput. 2011, 7, 625-632.

10. Humphrey, W.; Dalke, A.; Schulten, K. J. Mol. Graph. 1996, 14.

11. Case, D. A.; Babin, V.; Berryman, J. T.; Betz, R. M.; Cai, Q.; Cerutti, D. S.; III, T. E. C.; T.A. Darden; Duke, R. E.; Gohlke, H.; Goetz, A. W.; Gusarov, S.; Homeyer, N.; Janowski, P.; Kaus, J.; Kolossváry, I.; Kovalenko, A.; Lee, T. S.; LeGrand, S.; T. Luchko; Luo, R.; Madej, B.; Merz, K. M.; Paesani, F.; Roe, D. R.; Roitberg, A.; C. Sagui; Salomon-Ferrer, R.; Seabra, G.; Simmerling, C. L.; Smith, W.; Swails, J.; Walker, R. C.; Wang, J.; Wolf, R. M.; Wu, X.; Kollman, P. A. University of California, San Francisco, 2014.

12. (a) j. Wang, W. Wang, P. A. Kollman, D. a. Case, J. Mol. Graph. Mod., 2006, 25, 247-260; (b) J. Wang, R. M. Wolf, J. W. Caldwell, P. A. Kollman, D. A. Case, J. Comput. Chem., 2004, 25, 1157-1174.
